# Cost‐effectiveness analysis of a biopsy‐free diagnostic strategy for prostate cancer using mpMRI and PSMA‐PET/CT

**DOI:** 10.1002/bco2.70172

**Published:** 2026-02-04

**Authors:** Joshua Yi Min Tung, Rachel Shu‐En Lau, Edmund Pek Siang Teo, Kit Mun Chow, Julene Hui Wun Ong, Timothy Siliang Lu, Weiren Chen, Jingqiu Li, Xinyan Yang, Alvin Yuanming Lee, Yu Guang Tan, Kae Jack Tay, Sue Ping Thang, Winnie Wing Chuen Lam, Yan Mee Law, Henry Sun Sien Ho, Christopher Wai Sam Cheng, John Shyi‐Peng Yuen, Kenneth Chen

**Affiliations:** ^1^ Department of Urology Singapore General Hospital Singapore; ^2^ Data Science and Artificial Intelligence Laboratory Singapore General Hospital Singapore; ^3^ Department of Urology National University Hospital Singapore; ^4^ Health Services Research Unit Singapore General Hospital Singapore; ^5^ Department of Nuclear Medicine and Molecular Imaging Singapore General Hospital Singapore; ^6^ Department of Diagnostic Radiology Singapore General Hospital Singapore

**Keywords:** cost‐effectiveness, imaging and diagnostics, prostate biopsy, prostate cancer, PSMA PET/CT

## Abstract

**Introduction:**

Prostate‐Specific Membrane Antigen Positron Emission Tomography Computed Tomography (PSMA PET/CT) has shown a higher diagnostic accuracy in prostate cancer imaging when compared to conventional modalities, with improved sensitivity and specificity rates when combined with multiparametric prostate Magnetic Resonance Imaging (mpMRI). We evaluated the cost‐effectiveness of a combined PSMA PET/CT and mpMRI biopsy‐free diagnostic approach for clinically significant prostate cancer (csPCa).

**Method:**

A decision tree model was designed to compare two diagnostic strategies for csPCa in men with raised Prostate Specific Antigen (PSA)—the first with conventional mpMRI followed by transperineal prostate biopsy versus a second biopsy‐free, PSMA PET/CT plus mpMRI combined imaging strategy. We evaluated the impact of each strategy on costs and Quality‐Adjusted‐Life‐Years (QALYs). Willingness‐to‐pay thresholds were set at 1× and 3× Gross Domestic Product (GDP). One‐way sensitivity analysis and probabilistic sensitivity analyses were performed.

**Results:**

A combined mpMRI and PSMA PET/CT diagnostic strategy was 0.04 QALY more effective but SGD$4088.03 more expensive than the conventional mpMRI and biopsy strategy. The incremental cost‐effective ratio (ICER) was SGD$92782.87 per quality‐adjusted life year (QALY). In the combined imaging strategy, 3.5% of the cohort had a missed diagnosis of prostate cancer versus 13.9% in the conventional mpMRI and biopsy strategy. Probabilistic analyses showed that the combined imaging strategy was cost‐effective at willingness‐to‐pay thresholds of SGD$121160 and SGD$363480, respectively.

**Conclusion:**

Combined mpMRI and PSMA PET/CT for csPCa diagnosis are a cost‐effective strategy in terms of health utility over the conventional approach for diagnosing csPCa in men with raised PSA, potentially reducing the need for invasive diagnostic procedures.

## 1 INTRODUCTION

Prostate cancer is the second most frequently diagnosed malignancy among men and the fifth leading cause of cancer‐related mortality worldwide.^1^ Diagnostic pathways for prostate cancer have evolved substantially, due to the indolence of clinically insignificant prostate cancer.^2^, ^3^ Previous diagnostic pathways subjected men with elevated prostate specific antigen (PSA) or abnormal digital rectal examination to systematic prostatic biopsies, which often led to the detection of low‐grade, indolent cancers, whilst incurring costs and Quality‐of‐Life (QoL) decrements associated with invasive biopsy procedures. Although biopsy remains the gold standard for definitive diagnosis, it carries substantial risks of morbidity, including bleeding, infection and patient discomfort, thus raising interest in non‐invasive diagnostic approaches.^4^

The introduction of multi‐parametric MRI (mpMRI) as an initial triage test has improved the prostate cancer diagnostic pathway by enabling better risk‐stratification. Large trials such as the PRECISION and PRECISE trials have shown that using mpMRI as a triage test increases the detection of clinically significant prostate cancer (csPCa), whilst reducing over‐detection of clinically insignificant disease.^5^, ^6^ Despite these advances, mpMRI is not infallible—neither the sensitivity nor positive predictive value are perfect, leading to missed diagnoses if MRI alone is used as a triage test, and many men with MRI‐suspicious lesions ultimately have benign pathology or clinically insignificant disease.

Recent advancements in prostate‐specific membrane antigen (PSMA) positron emission tomography/computed tomography (PET/CT) have significantly improved the detection and characterization of prostate cancer lesions, demonstrating superior sensitivity and specificity compared to traditional imaging modalities such as CT, bone scans and mpMRI alone.^7^, ^8^ The landmark proPSMA randomized controlled trial demonstrated that PSMA PET/CT provided significantly higher diagnostic accuracy compared to conventional imaging modalities, which directly influenced clinical management decisions in a substantial proportion of patients.^9^ The PRIMARY study showed that the addition of PSMA PET/CT to mpMRI improved the detection of csPCa and reduced false‐negative rates.^10^ Subsequent studies have shown further promise in the use of PSMA PET/CT to accurately detect prostate cancer lesions at primary diagnosis, allowing for identification of clinically significant disease without reliance on invasive procedures.^11^, ^12^

The increasing accuracy of PSMA PET/CT raises the possibility of adopting a biopsy‐free diagnostic pathway when used in combination with mpMRI, potentially reducing procedural risks, patient discomfort and healthcare resource utilization.^13^ However, the cost‐effectiveness of such a strategy compared to standard biopsy‐dependent diagnostic pathways remains unclear. Whereas several economic evaluations have evaluated the cost‐effectiveness of PSMA PET/CT for staging purposes,^14^, ^15^, ^16^, ^17^ or for further stratification after an equivocal MRI,^18^ these analyses did not evaluate its application as a primary diagnostic modality in lieu of a biopsy.

The aim of this study was to conduct a cost‐effectiveness analysis comparing a combined mpMRI and PSMA PET/CT, biopsy‐free diagnostic strategy for csPCA, versus the conventional diagnostic strategy of mpMRI followed by biopsy.

## 2 METHODS

### 2.1 Model structure

We developed a decision‐analytic model in TreeAge Pro Healthcare 2025 (TreeAge Software, Williamstown, MA) to compare two diagnostic pathways for men suspected to have prostate cancer based on an elevated PSA between 10 and 20 ng/ml.

In the conventional diagnostic strategy arm, all patients received an mpMRI, followed by a confirmatory transperineal, MRI‐fusion guided prostate biopsy if the MRI was positive, defined as Prostate Imaging Reporting and Data System (PIRADS) 3–5 based on pre‐defined imaging criteria from studies included in Chow et al.'s^19^ meta‐analysis. Patients who had a positive MRI but negative initial biopsy could undergo repeat investigation comprising a repeat MRI, repeat biopsy or both, based on the real‐world natural history from our institution of such MRI‐positive lesions.

**FIGURE 1:**
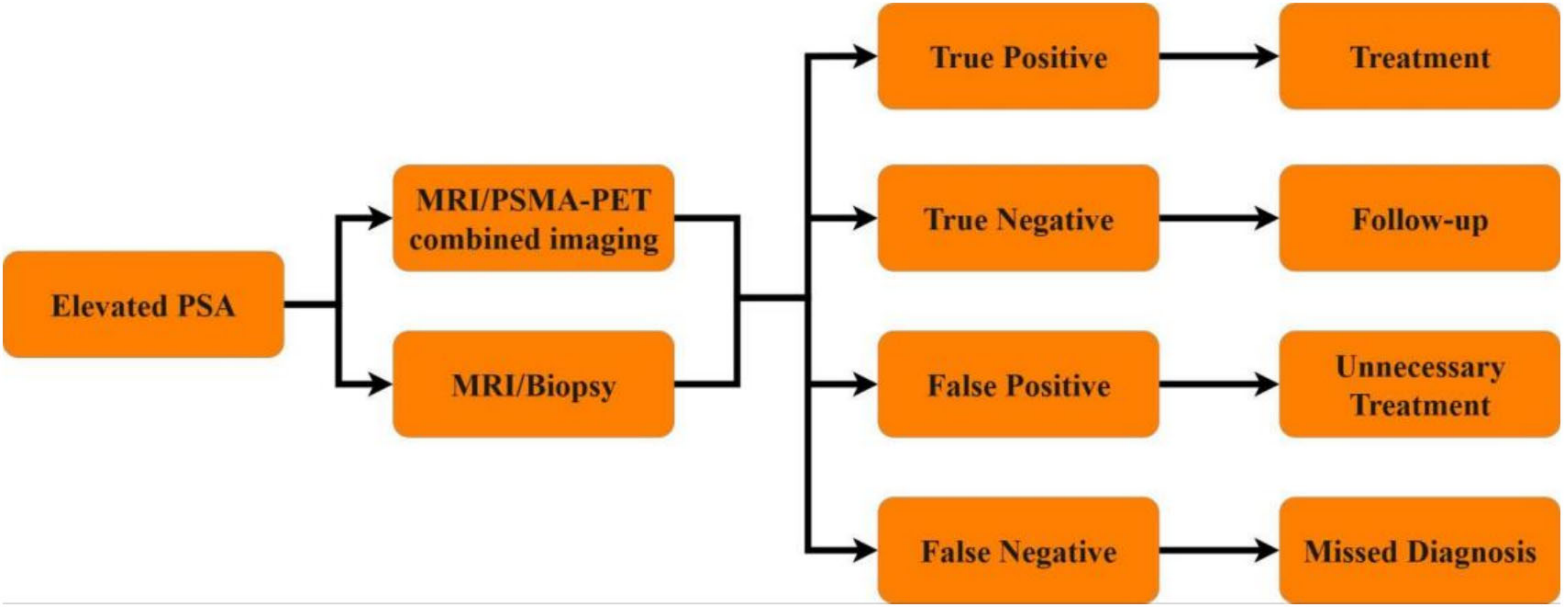
Diagrammatic representation of the model structure.

In the imaging‐only, biopsy‐free pathway, all patients received both an mpMRI and a PSMA PET/CT; if the combined imaging results were positive, the patient proceeded directly to definitive treatment without a confirmatory biopsy. If combined imaging showed no evidence of csPCA, the patient was managed with observation via clinical follow‐up and PSA monitoring. The analysis was conducted from the healthcare system perspective. Figure 1 shows the diagrammatic representation of the model structure.

### 2.2 Model input

Transitional probabilities, health utility values and costs are presented in Table S1.

### 2.3 Probabilities

Values for prevalence of csPCa in the simulated cohort, as well as the diagnostic accuracy of each strategy, were derived from existing meta‐analyses. The probability of post‐biopsy complications, stratified by Clavien‐Dindo score, was sourced from the European Association of Urology guidelines, which were themselves based upon results from the FUTURE randomized controlled trial. No Clavien‐Dindo Grade 3, 4 or 5 complications were recorded.

### 2.4 Health utility and costs

Health utility values were derived from existing literature. A healthcare payer's perspective was adopted for the model, and only direct unsubsidized costs were included. Costs were valued in Singapore Dollars (SGD) and based on typical bill sizes from a large tertiary specialist centre (Singapore General Hospital), as well as from published data by the Ministry of Health. Expected disutility and costs arising from procedural complications were calculated by multiplying the cost or disutility by the probability of the complication occurring.

### 2.5 Sensitivity analyses

We conducted deterministic one‐way sensitivity analyses (OWSA) and probabilistic sensitivity analysis consistent with CHEERS and ISPOR modelling guidance. For OWSA, each parameter was varied individually across prespecified ranges derived from 95% confidence intervals where available, or otherwise, clinically plausible bounds or known ranges (e.g., costs of surgery). Results are presented as a tornado diagram.

A probabilistic sensitivity analysis was also performed with 10 000 iterations. We assigned probability distributions to uncertain parameters, using Gamma distributions for costs and Beta distributions for probabilities and utility values. Results are presented as cost‐effectiveness analysis curves (CEACs).

## 3 RESULTS

### 3.1 Base‐case analysis

In the base‐case simulation comprising men with PSA between 10 and 20 ng/ml, the conventional diagnostic pathway of mpMRI followed by prostate biopsy yielded an expected average cost of SGD$20379.55 per patient and an expected average effectiveness of 0.92 QALY. In comparison, the combined mpMRI + PSMA PET/CT strategy had a higher upfront cost due to all patients receiving dual imaging, with an expected average cost of SGD$24467.58 per patient, but produced 0.97 QALYs on average. The combined imaging strategy thus incurred an incremental cost of SGD$4088.03 per patient, with an incremental QALY gain of 0.5 per patient versus standard care, resulting in an incremental cost‐effectiveness ratio (ICER) of approximately SGD$92782.87/QALY. Given a WTP threshold of SGD$121, 160 (1× GDP), the combined imaging strategy was cost‐effective. In absolute terms, however, the difference in QALYs between the two strategies was small, reflecting the fact that in the short‐term, most patients would do well in either pathway, and the harms of over‐treatment in the combined imaging arm would have been offset by the benefits of avoiding some biopsy‐related costs and QALY decrements.

### 3.2 Clinical outcomes

In the conventional pathway, by design, all men with a positive MRI proceeded on to transperineal prostate biopsy. This resulted in a 22.5% probability of the patient undergoing an ‘unnecessary’ biopsy procedure. At the same time, there was a 13.9% probability of a missed diagnosis. In contrast, the probability of a missed diagnosis using the combined imaging strategy was only 3.5%. This came at the cost of a 15.5% probability of the patient undergoing unnecessary treatment.

### 3.3 Sensitivity analyses

**FIGURE 2:**
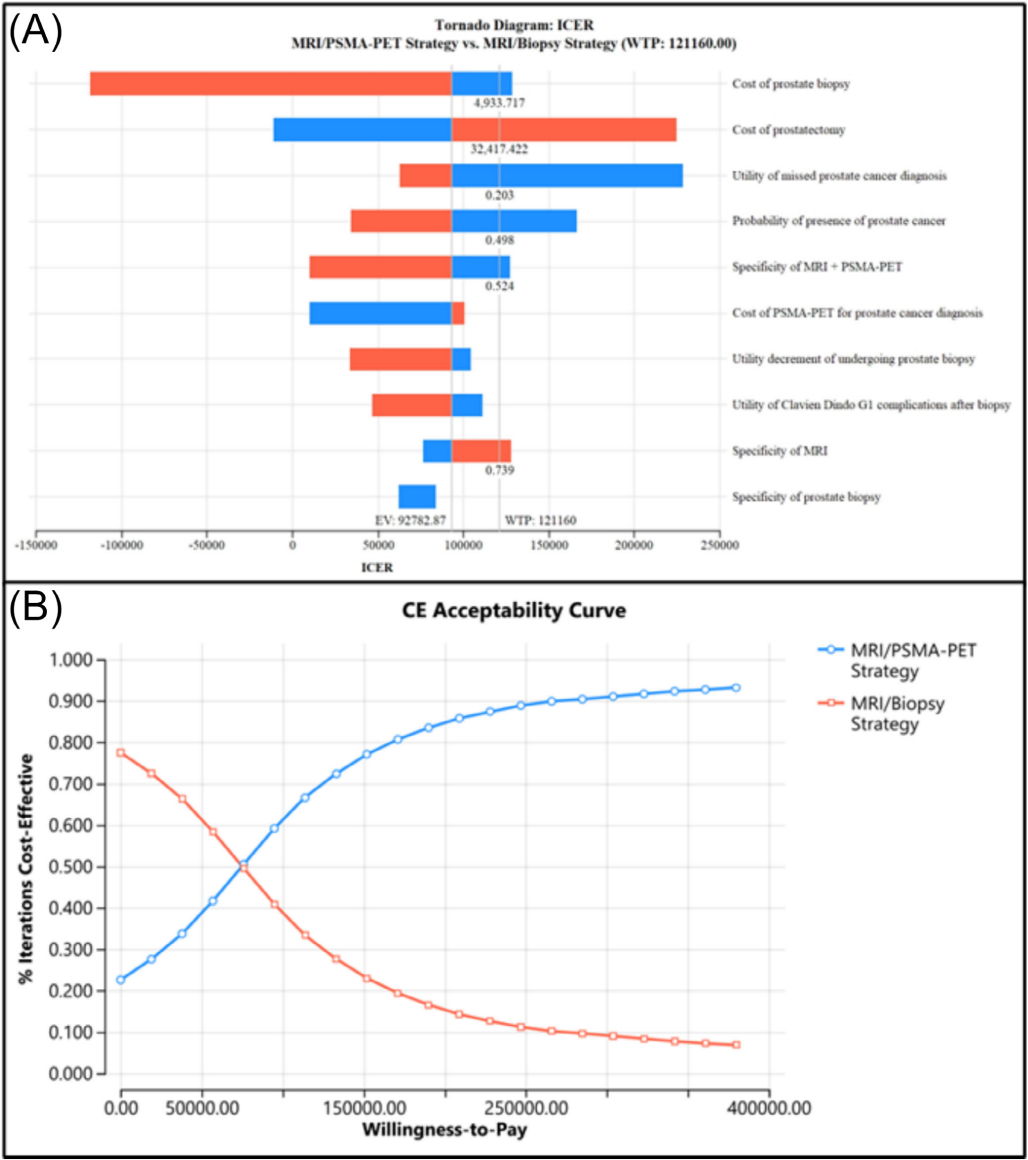
(A) Tornado diagram illustrating the most influential variables and levels at which the willingness‐to‐pay threshold is crossed and (B) cost‐effectiveness acceptability curve comparing the percentage of cost‐effective iterations of both strategies against willingness‐to‐pay thresholds.

One‐way sensitivity analyses indicated that the cost‐effectiveness of the combined imaging strategy was most sensitive to the cost of prostate biopsy, cost of csPCA treatment (prostatectomy), the health disutility of a missed csPCA diagnosis, the prevalence of csPCA in the included cohort and the specificity of both MRI alone and the MRI plus PMSA‐PET/CT combined imaging pathway. These parameters crossed the decision threshold, indicating potential for a switch in preferred strategy under these conditions. The tornado diagram in Figure 2A shows the most influential factors and the values at which the WTP threshold is crossed.

Across 10 000 simulations in the probabilistic sensitivity analysis, mean incremental outcomes for combined imaging versus conventional strategies were ΔCost = SGD$3553.81 and ΔQALY = 0.05. The CEAC shown in Figure 2B indicates a 68.9% probability that the combined imaging strategy is cost‐effective at SGD$121160/QALY, rising to 92.8% at SGD$363480.

## 4 DISCUSSION

Current guidelines recommend a risk stratification approach when investigating a patient for suspected prostate cancer based on investigations including PSA and MRI prostate if available, with subsequent consideration of performing a targeted prostate biopsy for histological confirmation prior to treatment of clinically significant prostate cancer.^20^

However, although MRI prostate is highly sensitive in identifying patients with csPCa, its specificity is poor, leading to unnecessary prostate biopsies, added costs and excess morbidity in MRI‐positive men without csPCA.^21^ Furthermore, with increased public awareness about early prostate cancer detection, and growing global trends towards PSA screening, the traditional pathway for diagnosing prostate cancer has led to an increasing demand for prostate biopsies.^22^ This can be resource intensive, requiring operating theatre, surgeon and anaesthetist availability. As such, resource limitations can lead to prolonged wait times with delays between time from presentation to diagnosis and ultimately to treatment.^23^

Our analysis found that a biopsy‐free, combined mpMRI + PSMA‐PET/CT imaging strategy can be cost‐effective when compared to the conventional mpMRI followed by prostate biopsy strategy. The principal drivers of cost‐effectiveness are biopsy avoidance, which saves on procedural and complication‐related costs and disutilities, and the higher sensitivity and specificity of combined imaging, which reduces the probability of a missed diagnosis or over‐diagnosis of clinically insignificant disease. Offsetting these gains is the risk of over‐treatment as a result of false‐positive imaging results, which carry non‐trivial cost and QoL penalties.

One‐way sensitivity analyses showed that with increasing prevalence of csPCa in the target population, the combined imaging strategy became more cost‐effective. At a population prevalence of 70%, the ICER dropped to SGD$56856.78, well below the WTP threshold. This implies that in a population of carefully selected men with a high suspicion of csPCa, a biopsy‐free diagnostic pathway could be even more cost‐effective.

Pre‐defined criteria for considering combined mpMRI and PSMA PET/CT imaging findings as ‘positive’ directly impact the specificity, and thus cost‐effectiveness, of this diagnostic strategy. The inclusion of PIRADS 3 lesions on mpMRI as ‘positive criteria’ in the PRIMARY and subsequent trials would have reduced the specificity of combined imaging. Analysis of the PRIMARY trial data shows that given a PIRADS 4 or 5 lesion on mpMRI, an SUVmax of 8.7 would have yielded a specificity of 100%.^24^ Two‐way sensitivity analyses from our model show that at a specificity of 95%, a reduction in cost of PSMA PET/CT imaging to SGD$3700 would result in absolute dominance of the combined diagnostic imaging strategy, with an ICER of ‐SGD$509.48.

### 4.1 Strengths

To the best of our knowledge, this is the first cost‐effective analysis of a biopsy‐free pathway using mpMRI and PSMA‐PET/CT for diagnosing prostate cancer. Our decision‐analytic model was developed based on existing data from meta‐analyses and further augmented with real‐world data for the natural history of patients who had a positive MRI but negative initial biopsy. Our sensitivity analyses reflected realistic implementation variants and market evolution, such as falling costs of PET imaging.

### 4.2 Limitations

Based on current literature, evidence on diagnostic thresholds for PSMA‐PET positivity, such as SUVmax cut‐offs, remains heterogeneous. Additionally, many studies classify MRI positivity as PIRADS ≥3, with limited data on stratified accuracy by PIRADS category. Our model therefore accepted broad inclusion criteria, which may dilute specificity. Narrowing the application of a combined‐imaging strategy to PIRADS ≥4 or PIRADS 5 only, in conjunction with high‐uptake lesions on PSMA‐PET could improve cost‐effectiveness but would reduce generalisability.

As a decision‐analytic model, a short time horizon was applied. Our model also focused on prostatectomy as the primary definitive treatment option for csPCa. A longer time horizon, alternative treatment modalities such as radiotherapy and/or focal therapy and associated utilities were not included and could shift value estimates. However, given the multitude of treatment options available and the associated plethora of possible branching post‐diagnosis management pathways, as well as the relative lack of strong real‐world evidence for transitional probabilities for each of these potential branches, a comprehensive modelling of management for csPCa was not undertaken in this analysis for which our focus was on diagnosis of csPCa. Lastly, our cost structure reflects our national context and may require re‐parameterization for other healthcare systems.

## 5 CONCLUSION

A biopsy‐free approach using mpMRI and PSMA PET is a cost‐effective pathway to diagnosing clinically significant prostate cancer. Value improves further with more careful patient selection, more stringent imaging thresholds, which raise specificity and lower PSMA‐PET costs; under favourable assumptions, this strategy could be cost‐saving. Prospective studies would be warranted to validate clinical safety and improve economic estimates.

## AUTHOR CONTRIBUTIONS

**Joshua Yi Min Tung**: Conceptualization; methodology; data curation; writing—original draft; writing—review and editing. **Rachel Shu‐En Lau**: Conceptualization; methodology; data curation; writing—original draft; writing—review and editing. **Edmund Pek Siang Teo**: Software; formal analysis; visualization. **Kit Mun Chow**: Conceptualization; methodology; writing—review and editing. **Julene Hui Wun Ong**: Data curation. **Timothy** **Siliang** **Lu**: Writing—review and editing. **Weiren Chen**: Writing—review and editing. **Jingqiu** **Li**: Writing—review and editing. **Xinyan Yang**: Writing—review and editing. **Alvin Yuanming Lee**: Writing—review and editing. **Yu Guang Tan**: Writing—review and editing. **Kae Jack Tay**: Writing—review and editing. **Sue Ping Thang**: Writing—review and editing. **Winnie Wing Chuen Lam**: Writing—review and editing. **Yan Mee Law**: Supervision. **Henry Sun Sien Ho**: Supervision; writing—review and editing. **Christopher Wai Sam Cheng**: Funding acquisition; supervision. **John Shyi‐Peng Yuen**: Funding acquisition; supervision. **Kenneth Chen**: Supervision; writing—review and editing.

## CONFLICT OF INTEREST STATEMENT

The authors declare no conflicts of interest.

## Supporting information


**Table S1.** Transitional probabilities, health utility values, and costs

## Data Availability

The datasets used and/or analysed during the current study are available from the corresponding author on reasonable request.

## References

[1] Siegel RL , Giaquinto AN , Jemal A . Cancer statistics, 2024. CA Cancer J Clin. 2024;74(1):12–49. 10.3322/caac.21820 38230766

[2] Lomas DJ , Ahmed HU . All change in the prostate cancer diagnostic pathway. Nat Rev Clin Oncol. 2020;17(6):372–381. 10.1038/s41571-020-0332-z 32112055

[3] Polascik TJ , Passoni NM , Villers A , Choyke PL . Modernizing the diagnostic and decision‐making pathway for prostate cancer. Clin Cancer Res. 2014;20(24):6254–6257. 10.1158/1078-0432.CCR-14-0247 25316814 PMC6330107

[4] Ahmed HU , El‐Shater Bosaily A , Brown LC , Gabe R , Kaplan R , Parmar MK , et al. Diagnostic accuracy of multi‐parametric MRI and TRUS biopsy in prostate cancer (PROMIS): a paired validating confirmatory study. Lancet. 2017;389(10071):815–822. 10.1016/S0140-6736(16)32401-1 28110982

[5] Kasivisvanathan V , Rannikko AS , Borghi M , Panebianco V , Mynderse LA , Vaarala MH , et al. MRI‐targeted or standard biopsy for prostate‐cancer diagnosis. N Engl J Med. 2018;378(19):1767–1777. 10.1056/NEJMoa1801993 29552975 PMC9084630

[6] Klotz L , Chin J , Black PC , Finelli A , Anidjar M , Bladou F , et al. Comparison of multiparametric magnetic resonance imaging‐targeted biopsy with systematic transrectal ultrasonography biopsy for biopsy‐naive men at risk for prostate cancer: a phase 3 randomized clinical trial: a phase 3 randomized clinical trial. JAMA Oncol. 2021;7(4):534–542. 33538782 10.1001/jamaoncol.2020.7589 PMC7863017

[7] Jochumsen MR , Bouchelouche K . PSMA PET/CT for primary staging of prostate cancer—an updated overview. Semin Nucl Med. 2024;54(1):39–45. 10.1053/j.semnuclmed.2023.07.001 37487824

[8] Chow KM , So WZ , Lee HJ , Lee A , Yap DW , Takwoingi Y , et al. Head‐to‐head comparison of the diagnostic accuracy of prostate‐specific membrane antigen positron emission tomography and conventional imaging modalities for initial staging of intermediate‐ to high‐risk prostate cancer: a systematic review and meta‐analysis. Eur Urol. 2023 [cited 2025 Apr 13;84(1). PMID: Available from: https://pubmed.ncbi.nlm.nih.gov/37032189/ 10.1016/j.eururo.2023.03.001 37032189

[9] Hofman MS , Lawrentschuk N , Francis RJ , Tang C , Vela I , Thomas P , et al. Prostate‐specific membrane antigen PET‐CT in patients with high‐risk prostate cancer before curative‐intent surgery or radiotherapy (proPSMA): a prospective, randomised, multicentre study. Lancet. 2020;395(10231):1208–1216. 10.1016/S0140-6736(20)30314-7 32209449

[10] Emmett L , Buteau J , Papa N , Moon D , Thompson J , Roberts MJ , et al. The additive diagnostic value of prostate‐specific membrane antigen positron emission tomography computed tomography to multiparametric magnetic resonance imaging triage in the diagnosis of prostate cancer (PRIMARY): a prospective multicentre study. Eur Urol. 2021;80(6):682–689. 10.1016/j.eururo.2021.08.002 34465492

[11] Liu Y , Niu S , Luan X , Zhang X , Liu J , Zhang J , et al. Can 18F‐PSMA‐7Q PET/CT replace prostate biopsy for the diagnosis of prostate cancer?—a single‐center retrospective study. Transl Androl Urol. 2023;12(1):83–89. 36760865 10.21037/tau-22-813 PMC9906103

[12] Sharma AP , Kumar R , Chauhan R , Ziauddin SA , Singh S , Singh H , et al. Accuracy of combined multi‐parametric MRI and PSMA PET‐CT in diagnosing localized prostate cancer: newer horizons for a biopsy‐free pathway. Eur J Hybrid Imaging. 2023;7(1):24. 10.1186/s41824-023-00182-5 37945775 PMC10635997

[13] Meissner VH , Rauscher I , Schwamborn K , Neumann J , Miller G , Weber W , et al. Radical prostatectomy without prior biopsy following multiparametric magnetic resonance imaging and prostate‐specific membrane antigen positron emission tomography. Eur Urol. 2022;82(2):156–160. 10.1016/j.eururo.2021.11.019 34887117

[14] Holzgreve A , Unterrainer M , Calais J , Adams T , Oprea‐Lager DE , Goffin K , et al. Is PSMA PET/CT cost‐effective for the primary staging in prostate cancer? First results for European countries and the USA based on the proPSMA trial. Eur J Nucl Med Mol Imaging. 2023;50(12):3750–3754. 10.1007/s00259-023-06332-y 37428216 PMC10547650

[15] de Feria Cardet RE , Hofman MS , Segard T , Yim J , Williams S , Francis RJ , et al. Is prostate‐specific membrane antigen positron emission tomography/computed tomography imaging cost‐effective in prostate cancer: an analysis informed by the proPSMA trial. Eur Urol. 2021;79(3):413–418. 10.1016/j.eururo.2020.11.043 33341285

[16] Song R , Jeet V , Sharma R , Hoyle M , Parkinson B . Cost‐effectiveness analysis of prostate‐specific membrane antigen (PSMA) positron emission tomography/computed tomography (PET/CT) for the primary staging of prostate cancer in Australia. Pharmacoeconomics. 2022;40(8):807–821. 10.1007/s40273-022-01156-4 35761117 PMC9300561

[17] Yee CW , Harvey MJ , Xin Y , Kirson NY . Cost‐effectiveness modeling of prostate‐specific membrane antigen positron emission tomography with piflufolastat F 18 for the initial diagnosis of patients with prostate cancer in the United States. Pharmacoeconomics. 2024;42(2):231–247. 10.1007/s40273-023-01322-2 37934376 PMC10811023

[18] Privé BM , Govers TM , Israël B , Janssen MJR , Timmermans BJR , Peters SMB , et al. A cost‐effectiveness study of PSMA‐PET/CT for the detection of clinically significant prostate cancer. Eur J Nucl Med Mol Imaging. 2025;52(9):3159–3169. 10.1007/s00259-025-07190-6 40072531 PMC12222398

[19] Chow KM , Lee A , Peh D , Tan YG , Tay KJ , Ho H , et al. Combined prostate‐specific membrane antigen positron emission tomography and multiparametric magnetic resonance imaging for the diagnosis of clinically significant prostate cancer. Eur Urol Oncol. 2025;8(5):1393–1405. 10.1016/j.euo.2025.04.017 40506357

[20] Cornford P , van den Bergh RCN , Briers E , van den Broeck T , Brunckhorst O , Darraugh J , et al. EAU‐EANM‐ESTRO‐ESUR‐ISUP‐SIOG guidelines on prostate cancer‐2024 update. Part I: screening, diagnosis, and local treatment with curative intent. Eur Urol. 2024;86(2):148–163. 10.1016/j.eururo.2024.03.027 38614820

[21] Drost F‐JH , Osses DF , Nieboer D , Steyerberg EW , Bangma CH , Roobol MJ , et al. Prostate MRI, with or without MRI‐targeted biopsy, and systematic biopsy for detecting prostate cancer. Cochrane Database Syst Rev. 2019;4(4):CD012663. 10.1002/14651858.CD012663.pub2 31022301 PMC6483565

[22] James ND , Tannock I , N'Dow J , Feng F , Gillessen S , Ali SA , et al. The Lancet Commission on prostate cancer: planning for the surge in cases. Lancet. 2024;403(10437):1683–1722. 10.1016/S0140-6736(24)00651-2 38583453 PMC7617369

[23] Sierocka A , Brzozowski S , Marczak M , Bednarek M , Kozłowski R . An analysis of waiting times for the diagnosis and treatment of patients with prostate cancer established by the requirements of the fast‐track cancer treatment pathway, taking into account treatment steps. Cancers. 2025;17(11):1842. 10.3390/cancers17111842 40507323 PMC12153762

[24] Liu J , Dunne J , Touijer KA , Perera M , Lawrentschuk N . The performance and role of PSMA PET scans in localised prostate cancer. Soc Int Urol J. 2025;6(1):10. 10.3390/siuj6010010

