# Supplementary material for: Cost‐effectiveness analysis of a biopsy‐free diagnostic strategy for prostate cancer using mpMRI and PSMA‐PET/CT
Source: BJUI Compass. 2026 Feb 4;7(2):e70172. doi: 10.1002/bco2.70172 (PMC12872110; doi:10.1002/bco2.70172)
Supplement: Supplementary file 1 — Table S1. Transitional probabilities, health utility values, and costs [file BCO2-7-e70172-s001.docx]

**Cost-effectiveness analysis of a biopsy-free diagnostic strategy for prostate cancer using mpMRI and PSMA-PET/CT**

**Supplementary Table S1**

| **Parameter** | **Base Case Value** | **Reference** |
| --- | --- | --- |
| *Probabilities* |  |  |
| Prostate cancer prevalence | 57.5% | Chow et al(1) |
| MRI sensitivity | 87.5% | Chow et al |
| MRI specificity | 47.1% | Chow et al |
| Biopsy sensitivity | 86% | Tu et al(2) |
| Combined imaging (MRI + PSMA-PET/CT) sensitivity | 94% | Chow et al |
| Combined imaging (MRI + PSMA-PET/CT) specificity | 63.5% | Chow et al |
| Clavien-Dindo Grade 1 complication after biopsy | 65.8% | Wegelin et al(3) |
| Clavien-Dindo Grade 1 complication after biopsy | 5.1% | Wegelin et al(3) |
| Repeat investigation after initial positive MRI but negative biopsy | 37.8% | Singapore General Hospital |
|  |  |  |
| *Costs (SGD$)* |  |  |
| Multiparametric MRI | $976 | Singapore General Hospital |
| PSMA-PET/CT | $4159 | Singapore General Hospital |
| Prostate biopsy | $5674 | Singapore General Hospital; Ministry of Health Fee Benchmarks(4) |
| Post-processing of MRI images for targeted biopsy | $477 | Singapore General Hospital |
| Staging scans for prostate cancer | $2193.5 | Singapore General Hospital |
| Anaesthetic clinic consultation | $177.3 | Singapore General Hospital |
| Urology clinic consultation | $129.6 | Singapore General Hospital |
| Accident and Emergency visit | $154 | Singapore General Hospital |
| Active voiding trial “fill and pull” procedure for acute retention of urine | $131.8 | Singapore General Hospital |
| Clavien-Dindo Grade 1 complication | $141.8 | Singapore General Hospital |
| Clavien-Dindo Grade 2 complication | $7717 | MOH hospital bill sizes for 2023, T60B Septicemia without catastrophic CC(4) |
| Prostatectomy | $27602 | Singapore General Hospital; Ministry of Health Fee Benchmarks(4) |
| Follow-up for prostate cancer including prostate specific antigen testing | $361.6 | Singapore General Hospital |
|  |  |  |
| *Utilities* |  |  |
| Prostate biopsy | -0.00577 | Hao et al(5) |
| Acute retention of urine | -0.01513 | Chughtai et al(6) |
| Clavien-Dindo Grade 1 complication | -0.01419 | Chughtai et al(6) |
| Clavien-Dindo Grade 2 complication | -0.04027 | National Institute for Health and Care Excellence UK (NICE) Guidance (7) |
| Follow-up for prostate cancer | -0.03 | Heijnsdijk et al(8) |
| Missed diagnosis of clinically-significant prostate cancer | -0.3 | Heijnsdijk et al(8) |
| Prostatectomy | -0.02333 | Heijnsdijk et al(8) |

**References**

1. Chow KM, Lee A, Peh D, et al. Combined prostate-specific membrane antigen positron emission tomography and multiparametric magnetic resonance imaging for the diagnosis of clinically significant prostate cancer. Eur Urol Oncol [Internet]. 2025 June 11; Available from: https://doi.org/10.1016/j.euo.2025.04.017

2. Tu X, Liu Z, Chang T, et al. Transperineal magnetic resonance imaging-targeted biopsy may perform better than transrectal route in the detection of clinically significant prostate cancer: Systematic review and meta-analysis. Clin Genitourin Cancer. 2019 Oct;17(5):e860–70.

3. Wegelin O, Exterkate L, van der Leest M, et al. Complications and adverse events of three magnetic resonance imaging-based target biopsy techniques in the diagnosis of prostate cancer among men with prior negative biopsies: Results from the FUTURE trial, a multicentre randomised controlled trial. Eur Urol Oncol. 2019 Nov;2(6):617–24.

4. Hospital Bills and Fee Benchmarks [Internet]. Ministry of Health. [cited 2025 Sept 25]. Available from: https://www.moh.gov.sg/managing-expenses/bills-and-fee-benchmarks/hospital-bills-and-fee-benchmarks

5. Hao S, Discacciati A, Eklund M, et al. Cost-effectiveness of prostate cancer screening using magnetic resonance imaging or standard biopsy based on the STHLM3-MRI study. JAMA Oncol. 2022 Nov 10;9(1):88.

6. Chughtai B, Rojanasarot S, Neeser K, Gultyaev D, Amorosi SL, Shore ND. Cost-effectiveness and budget impact of emerging minimally invasive surgical treatments for benign prostatic hyperplasia. J Health Econ Outcomes Res. 2021 May 6;8(1):42–50.

7. Transperineal biopsy for diagnosing prostate cancer [Internet]. NICE. [cited 2025 Sept 25]. Available from: https://www.nice.org.uk/guidance/dg54

8. Heijnsdijk EAM, Denham D, de Koning HJ. The cost-effectiveness of Prostate Cancer detection with the use of prostate health index. Value Health. 2016 Mar;19(2):153–7.
